# Transport in Proton Exchange Membranes for Fuel Cell Applications—A Systematic Non-Equilibrium Approach

**DOI:** 10.3390/ma10060576

**Published:** 2017-05-25

**Authors:** Angie L. Rangel-Cárdenas, Ger J. M Koper

**Affiliations:** Chemical Engineering, Delft University of Technology, 2629 HZ Delft, The Netherlands; A.L.RangelCardenas@tudelft.nl

**Keywords:** non-equilibrium, interfacial effects, transport properties, coupling effects, transport coefficient matrix, PEM fuel cell, proton conductivity, water permeability, hydrogen permeability, diffusivity, electro-osmotic drag, 82.47.-a

## Abstract

We hypothesize that the properties of proton-exchange membranes for fuel cell applications cannot be described unambiguously unless interface effects are taken into account. In order to prove this, we first develop a thermodynamically consistent description of the transport properties in the membranes, both for a homogeneous membrane and for a homogeneous membrane with two surface layers in contact with the electrodes or holder material. For each subsystem, homogeneous membrane, and the two surface layers, we limit ourselves to four parameters as the system as a whole is considered to be isothermal. We subsequently analyze the experimental results on some standard membranes that have appeared in the literature and analyze these using the two different descriptions. This analysis yields relatively well-defined values for the homogeneous membrane parameters and estimates for those of the surface layers and hence supports our hypothesis. As demonstrated, the method used here allows for a critical evaluation of the literature values. Moreover, it allows optimization of stacked transport systems such as proton-exchange membrane fuel cell units where interfacial layers, such as that between the catalyst and membrane, are taken into account systematically.

## 1. Introduction

Proton-conducting, polymer electrolyte membranes (PEM), play an important role in fuel cell applications as they serve not only the function of separation between the anode and cathode sides, but also act as a solid electrolyte allowing the transport of charge. The most common material used for these applications is Nafion™ (DuPont, Wilmington, DE, USA), which consists of a tetrafluoroethylene (TFE) backbone and perfluoroalkyl ether (PFA) side chains terminated in sulfonic acid groups [1,2]. The combination of the hydrophobicity of the backbone with the hydrophilicity of the sulfonic acid functional group in one macromolecule confers Nafion™ the properties necessary for this application.

Given the evident importance of PEM membranes, a plethora of studies on their different properties has been done over the last few decades. However, it proves difficult to reach a consensus on their meaning as research aimed at understanding the underlying phenomena describing the behavior of membrane properties is performed in many different ways and under different conditions which often do not match the reality of a membrane in an operating fuel cell. Within this framework, and from the very intrinsic interest in membrane performance, it would be advantageous to have a systematic approach for assessment that facilitates description and posterior optimization of the technology.

A powerful tool to study systems that are not in global equilibrium, such as electrochemical systems, is non-equilibrium thermodynamics (NET). This approach consists of a reformulation of the second law of thermodynamics in terms of entropy production [3]. In this formulation, originally proposed by Onsager [4], the entropy production is given by the product sum of conjugate fluxes, *J_i_*, and forces, *X_i_*, in the system. In his theory, each flux is defined as a linear combination of all forces related by a coefficient *L_ij_*. Onsager later proved that the relations between these coefficients were reciprocal: *L_ij_* = *L_ji_*. These coefficients are also known as Onsager’s coefficients. (See next section).

This theory provides a more accurate description of transport in systems where pressure, concentration, and temperature gradients exist as it also includes the coupling between these processes. Furthermore, it quantifies the entropy, or lost work, that is produced during transport [5]. The need to design systems that waste less work, such as fuel cells, makes this approach even more appropriate. However, despite the advantages that irreversible thermodynamics can offer, its use has been quite limited [2,3,5,6]. Kreuer et al. [2] did impressive work at using this approach to describe membranes for fuel cell applications; they provided an extensive survey on methods from simulation to experimentation and transport mechanisms as understood to the date. However, the formalism they propose is not entirely consistent as the number of independent driving forces has to equal the number of independent fluxes, which is not the case in their approach [2].

Here we present a transport coefficient matrix method (TCM) that allows a systematic approach to the problem and a literature survey of the properties considered meaningful for PEM fuel cell systems. We do so for a Nafion™ 117 type of membrane and our purpose is to demonstrate that the “contact resistance” information can be extracted from bulk values for different membrane properties and that this information is already available in experiments performed by others although not always accounted for. Solutions to eliminating contact resistances have been presented in earlier works for proton conductivity in bare membranes [7] and for the effect of mass transport at the interfaces on diffusivity and permeability [8,9]. However, in practice, contact resistances are always present and the values for bare membranes become less meaningful; it is in this scenario that interfacial effects become critical and need to be understood if our goal is to systematically quantify energy losses in a fuel cell as a means to achieve more efficient, cheaper devices. Moreover, the method offers the possibility to extend the study to stacked systems, thus also allowing the assessment of fuel cell stacks and the disentanglement of interfacial effects. Some authors have attempted to study the electrical and mass interfaces [1,10,11,12,13,14], but, as with membranes, there is no consensus on the whole picture and these phenomena are not yet fully understood.

## 2. Transport Coefficient Matrix Method (TCM)

Here, we shall model the membrane as a slab of ionomer, component 0, of thickness d and cross sectional area A. Thereby, we shall strictly follow the rules of non-equilibrium thermodynamics as laid down in the monograph of de Groot and Mazur [15] or the one of Kondepudi and Prigogine [16] albeit that we use molar quantities here. The membrane contains water, component 1, and hydrogen, component 2, that pass through the membrane. Due to the water, the ionomer dissociates and protons become available for transport of charge, component 3. This charge transport occurs by means of electrodes at the extremities of the membrane. We fix our frame of reference to the ionomer material of the membrane so that fluxes of water, hydrogen, and charge are transported through the membrane in the direction perpendicular to the surface area. As the ionomer itself is stationary in the frame of reference, the number of components in the system is three: the two neutral species, water and hydrogen, and the charge. Temperature is assumed to be constant so that we will be able to map closely onto the work of Kreuer et al. [2]. 

As soon as transport takes place through the membrane, dissipation occurs which is quantified by means of the entropy production defined by

(1) $$\dot{S}=\frac{1}{T}\int_{V}JXdV$$

where *T* is the temperature, **JX** the flux-force conjugate, and *V* is the control volume. The fluxes are organized in a row vector as

(2) $$J=\left(J_{w},\;J_{h},\;j\right)$$

with the water flux as the first component, the hydrogen flux as the second component, and the current density as the third component. Likewise, the thermodynamic driving forces are defined by the column vector

(3) $$X=\left(\begin{array}{c} -\partial_{x}\mu_{w,T}\\ -\partial_{x}\mu_{h,T}\\ -\partial_{x}\phi \end{array}\right)$$

with the chemical potential gradient of water and hydrogen, both at constant temperature, as the first and second component and the electric field as the third component. 

The fluxes and the driving forces are coupled and, considering the magnitudes of the driving forces, these may be considered as linear so that a relation exists as

(4) $$J^{T}=LX$$

where **L** is the matrix of transport coefficients. Onsager symmetry [4] applies, so that of the nine elements of the matrix, there are only six that are independent. Note that the transformation of the driving forces produces a column vector that is mapped to the fluxes by matrix transposition.

The experimentally accessible transport quantities are the volume flux J_V_, of liquid water, and of hydrogen J_H_. The latter is defined with respect to the aqueous volume rather than to the ionomer. The transformation **B**, expressed as **J’ = JB**, changes the original flux vector in the new flux vector **J’** = (J_V_, J_H_, j) and has components

(5) $$B=\left(\begin{array}{ccc} V_{w} & V_{h} & 0\\ -c_{h}V_{h}/c_{w} & V_{h} & 0\\ 0 & 0 & 1 \end{array}\right)$$

where *V_w_* and *V_h_* are the water and hydrogen volume, respectively; and *c_w_* and −*c_h_* are the concentrations of water and hydrogen. The new thermodynamic variables are, apart from the remaining electric field, the experimentally accessible total pressure gradient and the partial pressure of hydrogen at constant total pressure. The old driving forces can be transferred into the new driving forces using the same transformation, i.e., X=BX' where

(6) $$X'=\left(\begin{array}{c} -\partial_{x}p\\ -\partial_{x}p_{H}\\ -\partial_{x}\phi \end{array}\right)$$

The transformation to new fluxes and forces leaves the entropy production unchanged because $JX=J'B^{-1}BX'=J'X'$.

The new transport coefficient matrix can be obtained from the old one through $L'=B^{T}LB$, and is symmetric because of Onsager symmetry and hence has six elements. It is written as

(7) $$\left(\begin{array}{c} J_{V}\\ J_{H}\\ j \end{array}\right)=\left(\begin{array}{ccc} P_{V} & 0 & \sigma K\\ 0 & P_{H} & 0\\ \sigma K & 0 & \sigma \end{array}\right)\left(\begin{array}{c} -\partial_{x}p\\ -\partial_{x}p_{H}\\ -\partial_{x}\phi \end{array}\right)$$

where *P_V_* and *P_H_* are the water and hydrogen permeabilities through the membrane, respectively; *σ* is the proton conductivity; and, *K* is the electro-osmotic drag. The three main coefficients are on the diagonal of the matrix. The first to consider is the permeability defined as the coefficient relating volumetric flow to total pressure gradient, i.e.,

(8) $$P_{V}={-\frac{J_{V}}{\partial_{x}p}|}_{\partial_{x}p_{h}=0,\;\partial_{x}\phi =0}$$

Likewise, there is the hydrogen permeability defined by

(9) $$P_{H}={-\frac{J_{H}}{\partial_{x}p_{H}}|}_{\partial_{x}p=0,\;\partial_{x}\phi =0}$$

Note that this involves a hydrogen gradient in the absence of a total pressure gradient. The third main coefficient is the electric conductivity, canonically defined as

(10) $$\sigma ={-\frac{j}{\partial_{x}\phi }|}_{\partial_{x}p=0,\;\partial_{x}p_{H}=0}$$

The off-diagonal coefficients are known as cross-coefficients in the field of non-equilibrium thermodynamics. Of the three that can be defined, only one is known; the electro-osmotic drag defined as

(11) $$K={\frac{J_{V}}{j}|}_{\partial_{x}p=0,\;\partial_{x}p_{H}=0}$$

The other two coefficients are assumed to be small [3,17] and therefore we have taken them to be zero in Equation (7).

The transport coefficients defined above depend on the temperature and on the water content, apart from more material dependencies such as the porosity of the ionomer. One generally assumes these to be uniform across the membrane volume of the ionomer whereas it is generally known not to be the case [2]. Actually, one only determines averaged values by considering the volumetric flow and electrical current for a whole membrane as a function of the pressure and electric potential drops across the membrane. The values thus obtained are then corrected by the geometrically estimated cross sectional area and thickness. 

There is, in general, not enough information available about membrane inhomogeneities to warrant a proper model. The most important inhomogeneities are the surfaces that are in contact with the electrodes or at least with a material to hold the ionomer in place. A well-known phenomenon that falls in this category is the “contact resistance” between a metallic electrode and a bulk material. Following Bedeaux and Kjelstrup [3,5] a relatively successful, thermodynamically consistent, approach to describe interfacial inhomogeneities consists of considering the membrane to be composed of a uniform central “bulk” material between two thin interfacial layers. The fluxes are considered to be continuous through these layers and hence the “resistance” of the individual layers is additive, i.e.,

(12) $$L_{\mathrm{tot}}^{-1}=\frac{\delta_{\mathrm{right}}L_{\mathrm{right}}^{-1}+dL_{\mathrm{mem}}^{-1}+\delta_{\mathrm{left}}L_{\mathrm{left}}^{-1}}{\delta_{\mathrm{right}}+d+\delta_{\mathrm{left}}}$$

where **L^−1^** is the inverse of the transport coefficient matrix and represents the resistivity of the membrane. The thicknesses of the interfacial layers, *δ*_left/right_, are generally not known and remain integrated within the coefficient matrix of the layers. 

In the following we shall analyze the literature information on a standard ionomer membrane both on the bulk and on the interfacial coefficient values. 

## 3. Membrane Properties: A Literature Survey

Proton conductivity, water permeability and diffusivity, hydrogen permeability, and electro-osmotic drag of Nafion™ membranes for fuel cell applications are presented in this section. State of the art measurements and proceedings are summarized and discussed in order to give a global idea of where the technology stands and the shortcomings of the current methods. Here we attempt to show that the interfaces play a bigger role than attributed to them by disentangling their effects from existing results and why it is important to consider them in fuel cell engineering.

### 3.1. Proton Conductivity

In PEM analysis, membrane properties are usually discussed in terms of water content, *λ*, a quantity expressed as the molar ratio of water molecules to sulfonate groups (–SO_3_^−^). When referring to proton conductivity it should be noted that charge transport happens through a hydrated membrane, that is, through the water held by a polymer matrix. Hence, the mechanism is not the same as in bulk water because of the additional forces exerted by the polymer matrix and sulfonate groups [2,8,18,19]. Nevertheless, to understand proton transfer in acidic hydrated membranes, such as Nafion™ in PEM fuel cells, it is important to firstly understand the transfer mechanisms in bulk water. In general, there are two mechanisms that describe this phenomenon, namely structure diffusion and vehicle diffusion. Their relative prevalence in bulk water significantly differs from that in membranes albeit that it also depends on the water content.

The structure diffusion of protons, also known as the Grotthuss mechanism, refers to the transfer of protons by tunneling from one water molecule to the next via hydrogen bonding, which is not an actual movement of the ion through the solvent but a rearrangement of atoms; this mechanism is often referred to as “proton hopping” [2,12,20]. On the other hand, it should be noted that water has a high self-diffusion coefficient which has a contribution on the total proton conductivity as protonated water molecules, in the form of H_3_O^+^ or H_9_O^4+^; this phenomenon is known as vehicle diffusion and it has a contribution of approximately 22% to the total conductivity assuming that the diffusion coefficients of H_2_O and H_3_O^+^ are the same at Standard Temperature and Pressure (STP) conditions [2,21]. 

In a hydrated acidic polymer, the ionomer material most used for PEM fuel cells, two types of domain can be recognized: hydrophobic domains constituting the polymer backbone that grant the membrane its morphological stability; and hydrophilic domains that allow the proton conduction and consist of protonated sulfonate groups (–SO_3_H). This domain is described as well-connected through nanochannels even at a low water content. Hence, percolation in these membranes is very good because there are almost no dead-end pockets [2,22,23]. Moreover, a transition region has been identified between the hydrophobic and hydrophilic domains which is considered to be the consequence of the side-chain architecture of Nafion™. This region is believed to confer Nafion™ with its swelling characteristic as it has been suggested that there is a progressive side-chain unfolding with increasing water content. 

Water content can be seen as the hydration of the –SO_3_^−^ groups and can be related to widening of the nanochannels and increasing conductivity as the membrane becomes more hydrated. At medium to high values of hydration, ~10 < *λ* < 22, the excess protons are located in the center of the nanochannels where the water is bulk-like and, thus, the proton transfer is similar to the phenomena described above for aqueous solutions with structure diffusion prevalently occurring. However, as the degree of hydration decreases, the concentration of protons increases, which generates more proton-donor than acceptor sites; this fact creates a bias of the hydrogen bonds in the electrostatic field which in turn suppresses structure diffusion. Hence, at low water content the transport of protons is mostly due to vehicular diffusion [2].

The characterization of proton conductivity can be done by either creating a faradaic current, i.e., where there is mass transfer, or by inducing a non-faradaic current, i.e., no mass transfer; in the former case, a redox couple is used to generate electrons as in the case of running electrochemical cells, and in the latter, charge is induced at the electrode interface by an external electric field as it is done in techniques such as NMR or Electrochemical Impedance Spectroscopy (EIS). Commonly, proton conductivity is assessed by EIS, a technique that determines the resistance of a membrane by applying an oscillating electric potential and varying its frequency [11,12,13,23,24,25,26,27,28,29,30]. However, properties determined by EIS are averaged quantities and its interpretation usually involves assuming isotropy of the material. The conductance of a membrane can be quantified by performing experiments in different configurations, namely in-plane [11,12,13,23,24,26,27,28,29,30] and through-plane measurements [25]. In-plane measurements quantify the conductance in the length of the membrane, while through-plane measurements do so across its thickness. Studies have shown that in-plane measurements are preferred over through-plane measurements as it will be discussed later.

**Figure 1:**
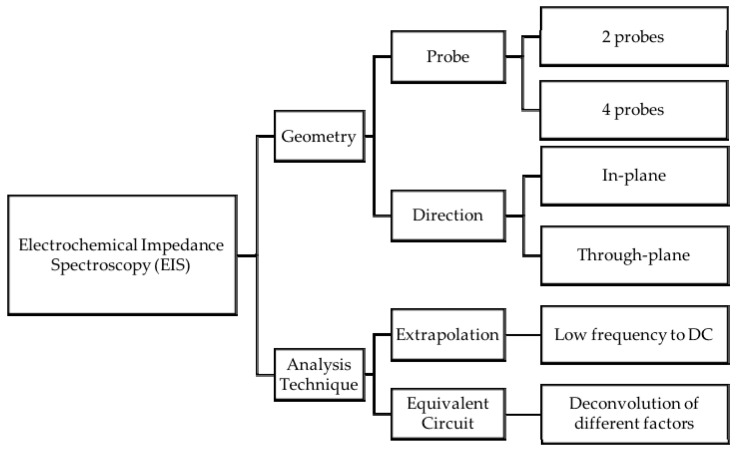
Electrochemical Impedance Spectroscopy: measurement and analysis techniques.

Reported conductivity data in the literature is often difficult to assess as results vary from laboratory to laboratory depending on experimental conditions. Studies have shown that conductivity measurements are influenced by the technique employed and the geometry of the conductivity cell [11,12,13]. In the first, conductivity values might be extracted by extrapolating the imaginary part of the measured impedance in the low frequency region [11,12,13,23,25,26,27,28,30] or extracted from fitting values for the components of an equivalent circuit [12,13]. In the latter, different geometries of the conductivity cell include a 2-probe [10,12,13,23,25,26,27,28] or 4-probe cell [12,13] where the distance between the measuring probes also plays a role. A summary of the possible configurations for EIS measurements is shown in Figure 1.

In the 2-probe configuration, the voltage measuring electrodes also carry the current. Under an alternating electric field and particularly at low frequencies, a certain amount of ions reaches the electrode before the reversal of polarity which results in charge build up at the interfaces and, thus, lowers the electric field in the bulk of the membrane; this phenomenon is often referred to as electrode blocking [12,13]. On the other hand, carrying out the impedance measurements using four probes helps diminish the effect of charge build up near the current carrying electrodes by using different electrodes sufficiently far away from the charge build-up region to measure the voltage across the bulk membrane material [12,13]. The voltage measuring electrodes are connected through a high impedance device so that the current flowing through them is negligible [12,13]. 

Conductivity measurements using the 4-probe method are appropriate for ionic conducting materials with low resistivity as the interfacial effects are diminished, whereas the 2-probe measurement is appropriate for high-resistance materials since other impedances present in the circuit can be neglected [12]. Furthermore, and regardless of the geometry of the cell, the subsequent analysis of the obtained data must be manipulated in order to retrieve the conductivity values. This is done either by extrapolating the obtained semicircle to its intercept with the real axis at low frequencies and taking this value as the bulk resistivity of the membrane; or, conversely, by fitting the obtained data to an equivalent circuit. In the latter, it is possible to disentangle the effects of the interfaces from those of the bulk material [12,13]. 

An important aspect that is often overlooked in the assessment of membranes is that the faradaic conductivity and the non-faradaic conductivity differ when the ionic species do not play the same role. In the case of conductivity measurements involving charge transfer at electrodes—in the faradaic setting—some ions are current carrying and others are blocked. Hence, it is possible to have excellent values for non-faradaic conductivity (no mass transfer) and extremely bad values in a faradaic setting (with mass transfer). For the present case of proton conducting membranes with anions fixed to the membrane, this is not expected to play a role as there is only one charge carrier which is also involved in the charge transfer at the electrodes.

**Figure 2:**
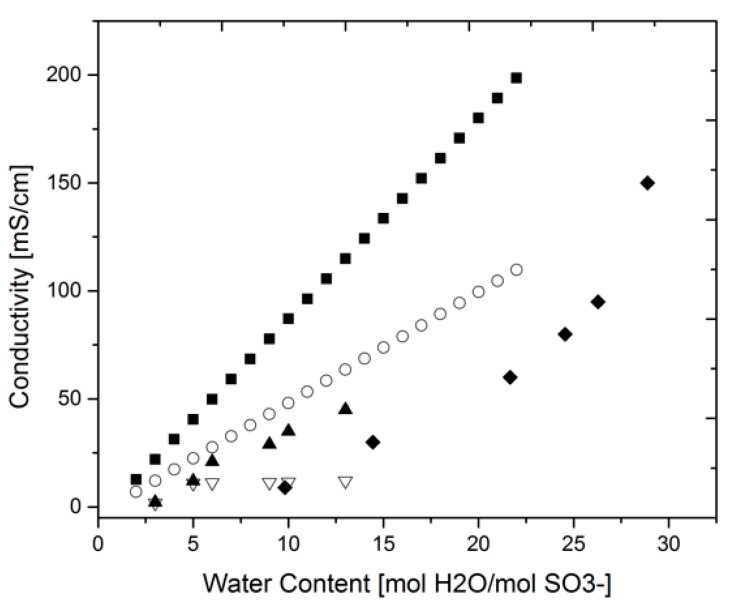
Nafion™ 117 conductivity value comparison. Springer et al. at 80 °C, in-plane, 2-probe (●) [30]; Zawodinski et al. at 30 °C, in-plane, 2-probe (○) [24]; Lee et al. at 60 °C, in-plane, 4-probe (▲) [12]; Lee et al. at 60 °C, in-plane, 2-probe (▽) [12]; Alberti et al., through-plane, 2-probe (♦) [25].

Despite the various different techniques with expected differing outcomes, it is found in the literature that conductivity measurements are done in many instances with disregard to the above mentioned potential errors. As a consequence, the lack of a standard measurement method and data analysis technique leads to varying results and imprecise estimations of the proton conductivity, which ultimately hinders the development of effective PEMs. In Figure 2 an example of different conductivity values for a bare Nafion™ 117 membrane found in the literature is presented. Even though the experiments were performed at different temperatures, it is evident that the values follow different trends for different measurement cell geometries. Zawodinski et al. [23,24] measured the proton conductivity at 30 °C in a 2-probe, in-plane set-up and later Springer et al. [10,18,30] correlated Zawondinski’s values for the water content and temperature according to

(13) $$\sigma \;[\mathrm{mS}/\mathrm{cm}]\;=\;\left(5.139\lambda -3.26\right)\exp\left\{1268\left(\frac{1}{303}-\frac{1}{T}\right)\right\}$$

The data and correlation from Zawodinski and Springer, respectively, have been widely used ever since as a benchmark [12]. Lee et al. [12] performed a systematic investigation on the effect of using two or four probes to measure conductivity in-plane at 60 °C. Their results showed a clear difference between the two methods indicating the nature of the contact effects as shown separately in Figure 2. However, their measured values are much lower than those of Zawodinski even though Lee et al. performed them at higher temperatures. This fact clearly shows the discrepancy found in the experimental results and the difficulty of assessing them properly. Reasons for this variability include the irreproducibility of the membranes, where no two samples are equal, e.g., different thickness, different pre-treatment method, in addition to the largely irreproducible effects of contacts or interfaces. This indeed calls for the necessity of standard measurement methods.

**Figure 3:**
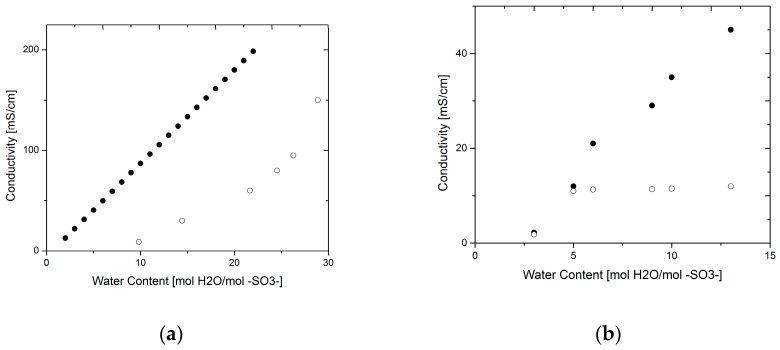
Effect of geometry: (**a**) Springer et al. [30] in-plane (●) vs. Alberti et al. [25] through-plane (○); (**b**) Lee et al. 2-probe (●) vs. 4-probe measurements (○) [12].

Figure 3 presents the differences in proton conductivity results obtained by the different geometries of the measuring cell. The data were fitted to an average function in both cases and the mean-square and root-mean-square errors were calculated; additionally, the percentage error was averaged over the whole range (see Section 4). A comparison of the 2-probe and 4-probe method yields an error of 34.6%, while the effect of the plane in which the measurement is done shows an error of 32.8%. These errors are a representation of the variability between measurements and support the hypothesis that the interfaces play a much more important role than is usually attributed to them. These effects are particularly notorious when comparing the results between the 2-probe and the 4-probe case, where the effect of the interfaces are known to play a role. At higher water content, where the Grotthuss mechanism of transport is dominant, the proton conductivity was lower for the 2-probe case due to other impedances present in the system apart from that of the bulk membrane. All the same, when comparing the plane in which the measurement was carried out, through-plane measurements yielded lower values which can be related to the fact that the interfaces, or area over which the current is being transported, is larger; hence, having a bigger contribution on the overall resistance. This fact is supported by studies performed at Scribner Associates Inc. labs where the contact resistance’s effect or “cell resistance” was eliminated by extrapolating the resistance at high frequencies to zero membrane thickness in a through-plane configuration. By doing this correction, they found that the membrane conductivity was the same for in- and through-plane measurements. This also provides evidence for the intrinsic isotropy of the material although this fact holds only for bare or untreated membranes as MEA (membrane electrode assembly) preparation processes such as hot-pressing may induce structural changes that affect charge transport in different directions [7].

### 3.2. Water Permeability

Water transport in proton-conducting membranes is of the most importance as water is mainly responsible for the transport of charges across the membrane. Therefore, the permeability of the membrane to water molecules and their diffusivity are properties that have been extensively studied [1,8,9,22,23,24]. The water permeation process through a membrane can be expressed in terms of three different steps, namely (i) sorption of water into the membrane at the sorption side; (ii) diffusion of water across the membrane; and (iii) desorption of water from the membrane at the dry side [8,9]. The sorption and desorption steps represent interfacial resistances to mass transport and have been studied by several authors [1,8,9]. From their permeation studies, they have reported that reasons for this resistance to be significant include the membrane’s surface being hydrophobic to water vapor and hydrophilic to liquid water. This has been proven by SAXS (Small Angle X-ray Scattering) experiments that corroborated structural changes of the membrane’s interface according to the medium it was in contact with [8]. Majsztrik et al. reported that the rate limiting step in permeation experiments was water desorption at the membrane/gas interface [9].

There are various methods to estimate water permeability and diffusivity (see next subsection) through a membrane [8] and unfortunately literature results are found to vary with the measuring technique. Zhao et al. and Majsztrik et al. have reviewed them and shown that results for permeability and diffusivity vary up to three orders of magnitude [8,9]. Water permeability is typically measured with a simple permeation experiment where two water-filled chambers are separated by the membrane and a total pressure difference is applied. The change in volume in the lower pressure chamber is measured with a capillary and related to the permeability of the membrane [2,31]. Alternatively, water diffusivity through the membrane can be measured by NMR [1,8,9,22,24] and then related to the permeability using the following argument. 

From Fick’s law, that relates mass flux to the concentration gradient, one obtains an expression in terms of the water diffusivity *D_w_* as

(14) $$J_{w}=-D_{w}\frac{\Delta c_{w}}{\delta }=-\frac{D_{w}c_{w}}{RT}\frac{\Delta \mu_{w}^{\left(l\right)}}{\delta }=-\frac{D_{w}c_{w}}{RT}\frac{\Delta \mu_{w}^{\left(v\right)}}{\delta }=-\frac{D_{w}}{p_{w}V_{w}}\frac{\Delta p_{w}}{\delta }$$

where *p_w_* is the water vapor pressure in equilibrium with the water in the membrane. The water permeability defined in the previous section is then related to the diffusivity as *P_w_* = *P_V_/V_w_* = *D_w_*/(*p_w_V_w_*) assuming that the experiments are done in the absence of hydrogen so that the volume flow only involves water. The water permeability is usually given in mol cm^−1^ s^−1^ bar^−1^ and the diffusivity in cm^2^ s^−1^.

**Figure 4:**
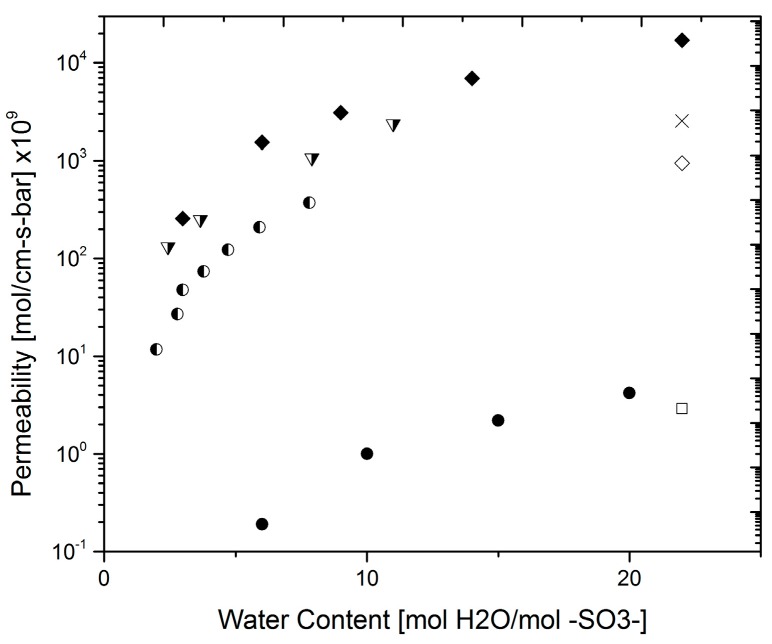
Water permeability. Ise, Kreuer at 27 °C (●) [31]; Rivin et al. at 32 °C (◻) [1]; Zhao et al. at 70 °C (◊) [8]; Majztrik et al. at 80 °C (×) [9]; Zawodinski et al. at 30 °C (♦) [23]; Zhao et al. at 70 °C (◐) [8]; Edmondson et al. at room temperature (⧩) [22].

Data gathered for permeability and diffusivity of water through a Nafion™ 117 membrane are shown in Figure 4. For comparison, the diffusivity values were converted to permeability using the above argument. The data were fit to an average function to assess the variability among experiments.

The data are spread over several orders of magnitude, indicating underlying phenomena that were not accounted for. The highest values correspond to those measured by NMR for the intra-diffusion coefficient; however, those obtained by permeation experiments are much lower. The data from permeability measurements and the calculated values from diffusivity seem to follow the same trend which is expected from the above argument; nevertheless, at low water contents the trend seems to be different. 

Two regimes can be identified in the permeability of the membrane, with a faster increase after a water content higher than 14. However, in the case of diffusivity there seems to be an inflexion point at a lower water content (~4). Zhao et al. [8] performed diffusivity experiments at different temperatures and the results followed the same trend. The change in regime in both cases suggests an interfacial effect as the resistance to mass transport at the liquid-liquid interface becomes lower with increasing amounts of water in the membrane; with more water the nanochannels swell, thus creating more space for water to move across [8,9]. An activation volume has been reported by various authors, where after a certain level of hydration of the membrane, its properties change at a different rate [22]; this activation volume or percolation threshold has been reported to occur at low water volume fractions (~0.005) [22]. At this point the membrane has enough water to connect the nanochannels, thus increasing percolation.

### 3.3. Hydrogen Permeability

**Figure 5:**
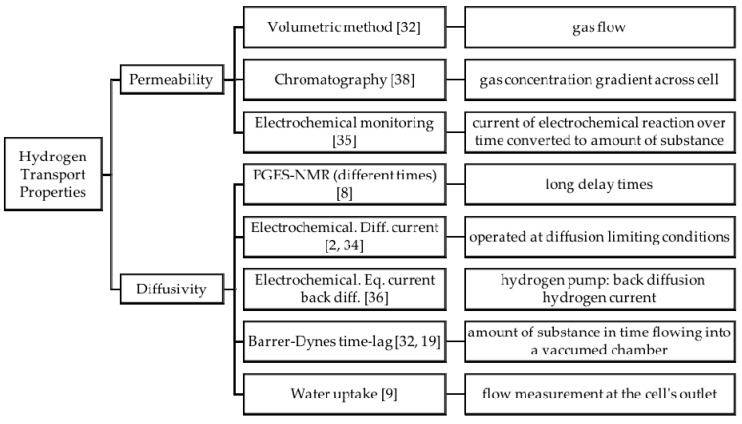
Permeability and Diffusivity measuring techniques.

One important issue present in proton-conducting membranes for PEM fuel cell applications is their permeability to hydrogen gas. It is important that the membrane is permeable to water as this provides the means for charge transport; however, this also means that hydrogen gas from the anode feed can move through the membrane as it dissolves in water. In turn, this results in fuel losses and hence efficiency losses. Due to this cross-over effect, the hydrogen mass transport properties have been given some attention in the past few decades [8,9,19,32,33,34,35,36,37]. Typical methods for permeability and diffusivity measurements are shown in Figure 5 (applicable for gases, e.g. oxygen and hydrogen, and water).

**Figure 6:**
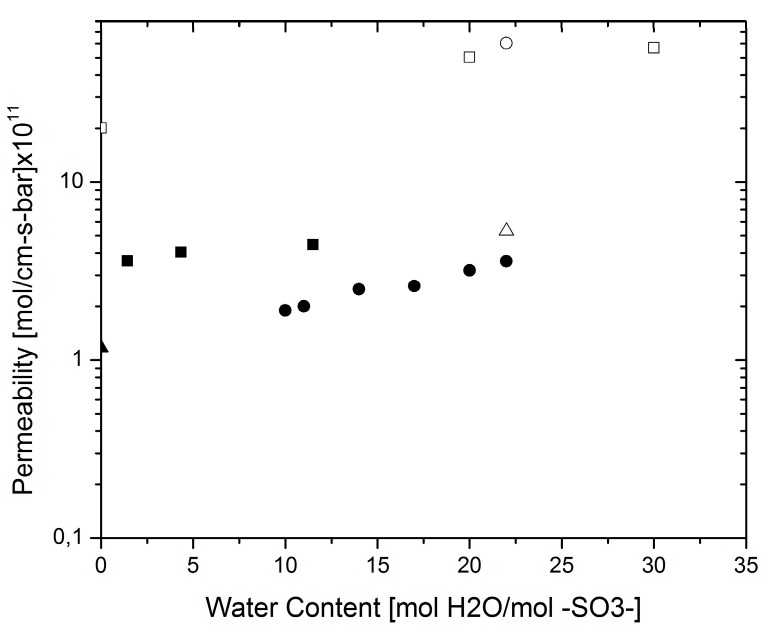
Hydrogen permeability. Schalenbach at 80 °C (●) [35]; Broka et al. at 80 °C (◻) [38]; Sakai et al. at 80 °C (◻) [32]; Jiang et al. at 80 °C (extrapolated) (○) [34]; Dry membrane (▲) [35]; Wet membrane (△) [35].

The preferred methods for hydrogen transport measurements are chromatography and electrochemical monitoring. Attempts were made by Sakai et al. and Wu et al. to use the Barrer-Dynes time-lag technique [19,32] but the diffusion time of hydrogen is too small to be measured by this technique. In order to make the results comparable, the mass transport will be assessed in terms of the permeability (mol cm^−1^ s^−1^ bar^−1^). Permeability and diffusivity results are interchangeable via the solubility of hydrogen in the membrane [32,33]; the permeability has been extracted from results reported as permeation flux (mol cm^−2^ s^−1^) by means of the hydrogen saturation pressure in water. Figure 6 shows different results from various measuring techniques for hydrogen permeability; for comparison, the points corresponding to a wet and a dry membrane are also shown. In this case, the variability was evaluated between experiments performed by an electrochemical technique [35] vs. chromatography [32] as the sample of data is spread over a larger range of water content values. The average relative error in this case was estimated to be ~28%. The higher values are those reported by Broka et al. [38] who used a volumetric method to measure hydrogen permeability, whereas electrochemical methods presented lower, but varying, values. This difference could be attributed to the presence of electrical interfaces in the electrochemical measurements. In these cases, protons were allowed through the membrane and the current generated was related to the amount of substance through Faraday’s law. Due to the electrical resistances at the interfaces, it is natural to assume electrical losses and thus, the measurements would yield lower values. This effect will be discussed further in the next section.

### 3.4. Electro-Osmotic Drag

**Figure 7:**
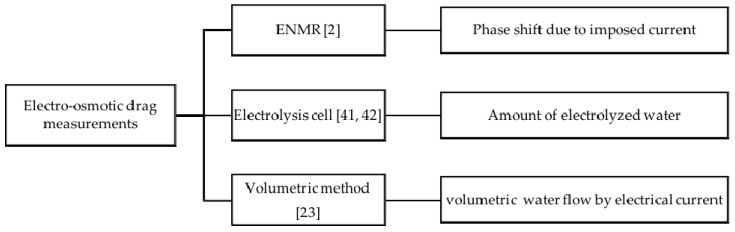
Electro-osmotic drag measuring techniques.

The motion of water is not only caused by the chemical potential difference, but also by the motion of protons, which is in return proportional to the current flowing through the membrane. This coupling effect is known as electro-osmotic drag and it is defined as the number of water molecules dragged per charge carrier [2,6,14,18,23,24,39,40,41,42]. Measuring this property is non-trivial and as a consequence it has not been widely studied. However, there have been some attempts in the literature which in turn might give some insights about the role the interfaces are playing. A summary of the methods used to measure the electro-osmotic drag to our knowledge is presented in Figure 7. 

**Figure 8:**
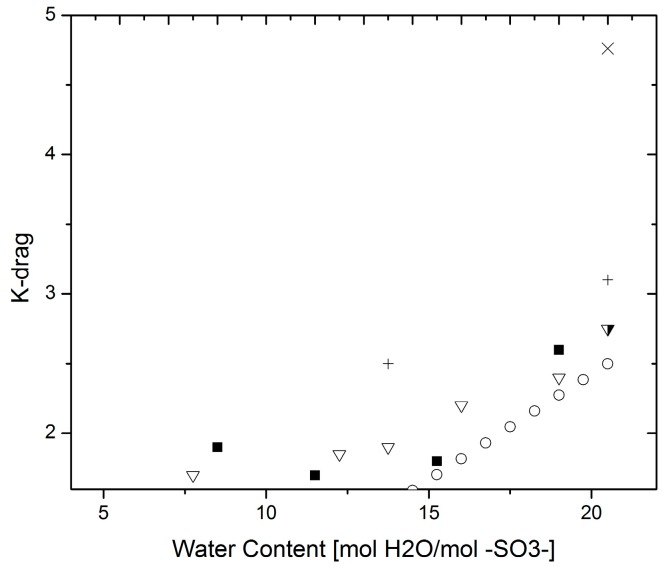
Electro-osmotic drag data compilation. Ise, Kreuer at 27 °C (◼) [31]; Kreuer et al. at 27 °C (▽) [40]; Springer et al. at 80 °C (○) [30]; Onda et al. at 80 °C (×) [42]; Kreuer et al. at 80 °C (+) [2]; Zawodinski at 30 °C (⧩) [23].

Figure 8 shows a compilation of data results that were rendered reliable for the analysis presented in this paper. The majority of the data have been gathered by Kreuer et al. over the course of years [2,31,40,43], gathering information at various temperatures and different water contents using electrophoretic NMR (ENMR), a set-up that was built in their group for this purpose. 

**Figure 9:**
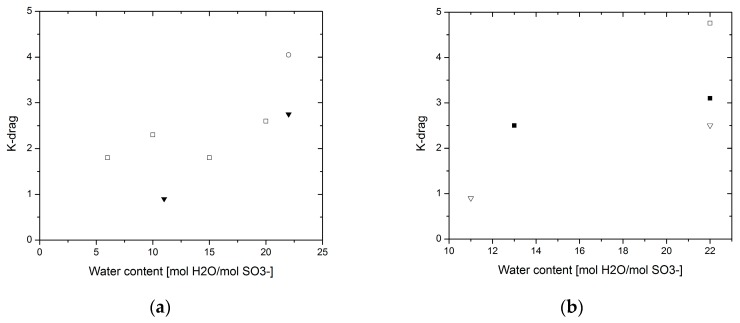
K-drag—(**a**) at 30 °C (**◻**) Electrophoretic NMR [43]; (▼) Volumetric method [23]; (○) Electrolysis cell [42]; (**b**) at 80 °C (**◻**) ENMR [40]; (▽) Volumetric [30]; (**◻**) Electrolysis cell [42].

A comparison between results obtained by different techniques at 30 °C and 80 °C gives errors of about 27.6% and 30.2%, respectively (calculated as indicated in the Proton conductivity section). The information at low water contents is very limited, but regardless of this fact, some information can be extracted if looking at a common point among the three techniques. In Figure 8 and Figure 9, it can be seen that at a water content of 22 mol H_2_O/mol –SO_3_^−^ the electro-osmotic drag values found were considerably different for each technique. The highest value was found by Onda et al. [42] where an electrochemical technique was employed for this purpose, whereas the values found by Kreuer et al. were measured by ENMR. The nature of these differences could be due to the effect of electric interfaces interfering with the measurement, however more evidence is necessary to support this hypothesis. 

## 4. Discussion 

In general, the assessment of membranes is not standardized which creates a chaotic amount of information that is difficult to assess and not entirely reliable as shown in the previous section. The data must be treated with special care and attention to the conditions and measuring techniques that were used, as these reveal information about the suitability of property values for certain applications. For instance, the proton conductivity measured with two probes in Figure 3 reflects mainly the resistances at the interfaces, whereas the measurement with four probes mainly reflects the bulk. For membrane science and development, the bulk measurement would be of most interest while in fuel cell engineering through-plane measurements would be more meaningful as a fuel cell operates in this configuration.

To illustrate this point further, the TCM methodology explained in Section 2 may be used to disentangle the effects of the bulk and interface for all properties. As proton conductivity remains constant after *λ* ~ 5 when measured with two probes (see Figure 3b), one may argue that this value reflects the combined effects of the bulk and interfaces as the resistances were not further decreasing with increasing water content, whereas the tendency of conductivity to increase with the water content in the 4-probe case suggests values closer to the bulk. From Equation (12), and considering the electro-osmotic drag to be negligible, it follows that

(15) $$\frac{\delta_{m}}{\sigma_{m}}=\frac{2\delta_{if}}{\sigma_{if}}+\frac{\delta_{b}}{\sigma_{b}}$$

where the suffixes *m*, *if*, and *b* stand for the membrane, interface, and bulk, respectively. Studies have shown that the extension of the interface in a Nafion™/Pt catalyst layer system is of a few nanometers (~5 nm) [44]. Considering that a standard Nafion™ 117 membrane has a dry thickness of 186 μm, the total value of the membrane conductivity can be computed. Equation (15) can be written for the 2-probe (*2p*) and 4-probe (*4p*) cases, as follows:

(16) $$\{\begin{array}{c} \frac{d_{m}}{\sigma_{2P}}=\frac{2d_{if}}{\sigma_{if}}+\frac{d_{b}}{\sigma_{b}}\\ \frac{d_{m}}{\sigma_{4P}}=\frac{d_{b}}{\sigma_{b}} \end{array}$$

where the subindexes *2p* and *4p* refer to the 2-probe and 4-probe measurements, respectively. Bulk, interface, and overall values can be computed by solving this simple set (see Table 1).

The same logic can be followed for water permeability, hydrogen permeability, and electro-osmotic drag. In the case of water permeability, Zhao et al. [8] characterized the water diffusivity by NMR, and subsequently calculated the permeability by correcting for the interfacial effects, i.e., based on varying water activities at the feed side, they estimated an interfacial mass transport coefficient as suggested in previous studies by Majzstrik et al. [9]. Therefore, the results found by Zhao et al. are approximated to be the bulk values, whereas those of Ise and Kreuer [31] found by a permeation experiment are considered to include large interfacial contributions. Consequently a corrected overall value for water permeability can be found from a procedure analogous to Equations (15) and (16).

Schalenbach et al. [35] measured hydrogen permeability using an electrochemical technique. In their experiment, hydrogen is left to permeate through a membrane followed by an electrochemical cell that converts the permeated hydrogen into protons; the generated current thus represents the permeation of hydrogen through the membrane and it is related to the total amount of gas by Faraday’s law. One may argue that due to the nature of the experiment, the overpotentials at the interfaces will consume a certain part of the voltage drop and hence the value that is being measured is lower than the actual charge transport due to permeability. If the permeability of hydrogen measured with a volumetric method [32] is assumed to have negligible interfacial contributions i.e., mainly bulk, and the electrochemical measurement to induce large losses at the interfaces, a similar treatment to that mentioned above applies in order to calculate a corrected overall hydrogen permeability of the membrane.

Similarly, for the electro-osmotic drag, the results by Zawodinski et al. [23] found by a volumetric method can be attributed to the bulk, whereas the measurements by Onda et al. [42] in an electrolysis cell would account for combined interfacial and bulk contributions. A summary of the overall property values is shown in Table 1.

**Figure 10:**
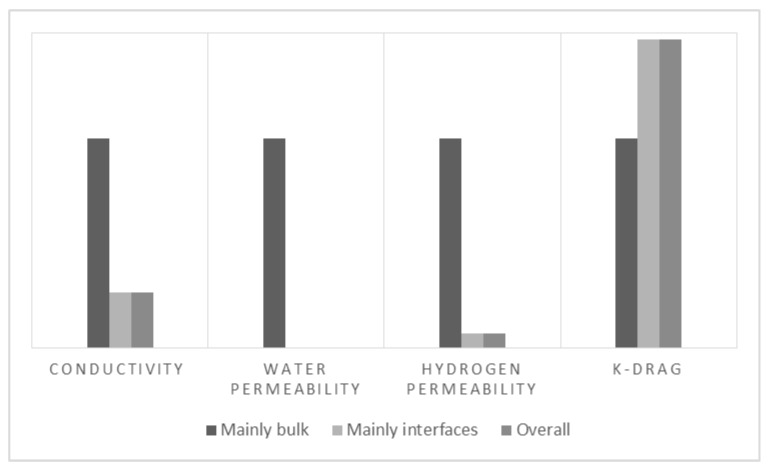
Comparison: measurements where there are mainly bulk contributions against measurements where mainly interfacial effects are present as compared to corrected overall values.

The corrected overall values show large deviations from measurements where the resistance contributions have been corrected for, and small differences with those where interfacial effects are thought to predominate. This behavior depicts the fact that the role of contact resistances becomes increasingly important in measurements where conditions resemble more closely those of an operating fuel cell. A normalized comparison and deviation percentages are shown in Figure 10 and Table 2.

When corrected for interfacial contributions, the overall proton conductivity equals that from the 2-probe measurement where interfacial effects are thought to predominate and differs 73% from those where the set-up has been improved to reduce contact resistances (4-probe). Even though 4-probe measurements are the main geometry used to assess proton conductivity, the large difference with the overall value indicates that this property will be significantly lower when the membrane is put into application. Similarly, water and hydrogen permeability show errors larger than 90% when corrected as compared to those where interfacial effects have been eliminated. This suggests that mass transfer at the interfaces is the limiting step. On the other hand, the electro-osmotic drag shows an opposite behavior due to the nature of the experiment where the amount of molecules carried by the current can be easily overestimated by the amount of electrolyzed water.

In general, these results highlight the necessity to assess membranes and MEAs in conditions as close as possibly to those of an operating fuel cell, as measurements of bare membranes can be highly deceptive. A large part of the research on transport properties has focused on isolating bulk values, whereas less attention has been given to the role of the interfaces on the overall results. Nevertheless, a few studies have demonstrated a deviation of certain properties in bulk membranes with respect to thin films where the behavior is expected to resemble the interfaces due to confinement effects [45,46]. 

Here, it has been shown that the effect of interfaces can be unraveled using the transport coefficient matrix (TCM) method and their effect over the total values for properties of interest in fuel cell engineering should be treated carefully, as it was made evident by the variability of the results found in the literature (see Table 3) and the large deviation of the overall values from the bare membranes. This fact supports the hypothesis that the interfaces play a greater role than commonly considered and highlight the necessity to further study interfaces in order to unravel and account for mechanisms of charge and mass transport if more efficient fuel cells are to be attained.

## 5. Conclusions

A systematic method to discriminate between bulk and interfacial resistances to mass and charge transport has been presented and exemplified for key membrane properties in PEM fuel cell applications. It was shown that this method allows unraveling of the interfacial effects and that this can be done from existing experimental results if critically assessed and handled. The proper handling of data highlights the necessity to carefully choose and understand experiments when measuring bare membranes or MEAs to closely resemble a fuel cell.

Interfacial effects on mass and charge transport resistances are crucial in fuel cell engineering and including rather than eliminating them is of prime importance for fuel cell development. This is supported by the results of our analysis where interfacial effects account for significant percentages of the total measured values, particularly in permeability measurements. This suggests that studying membranes as close as possible to fuel cell operation would yield more efficient and possibly cheaper fuel cells, and knowing the effects of contact resistances on all relevant properties is a powerful tool for optimization in this scenario.

Furthermore, it was shown that the results reported in the literature are of an erratic nature often due to the method in which properties are measured. This fact creates a difficulty in assessing and comparing them which ultimately results in hindering the progress of fuel cell engineering. The TCM method herein demostrated also offers a systematic way of presenting and analyzing the different properties and parameters deemed relevant for a certain system which could be advantageous for membrane and fuel cell studies by helping scientists and engineers gather more relevant and consistent data in a simpler, more accesible manner.

## Figures and Tables

**Table 1:** Summary of interfacial contributions.

Transport Coefficient	Bulk	Interface	Overall
Conductivity (mS/m)	45	0.88 × 10^−3^	12
Water permeability (mol/cm-s-bar) × 10^9^	948	2.3 × 10^−4^	4.2
Hydrogen permeability (mol/cm-s-bar) × 10^11^	50	2 × 10^−4^	3.6
Electro-osmotic drag (molecule H_2_O/charge carrier)	2.75	4.6 × 10^−4^	4.05

**Table 2:** Relative deviation of the overall values.

Transport Coefficient	Deviation from Bulk
Conductivity (mS/m)	−73.3%
Water permeability (mol/cm-s-bar) × 10^9^	−99.5%
Hydrogen permeability (mol/cm-s-bar) × 10^11^	−92.8%
Electro-osmotic drag (molecule H_2_O/charge carrier)	+47.2%

**Table 3:** Summary of errors between measurements.

Transport Coefficient	Conditions	RMSE (unit)	% Error
Conductivity (mS/cm)	2- vs. 4-probe	9.3	35%
	In- vs. Through-plane	41.2	33%
Water permeability (mol/cm-s-bar) × 10^9^	-	3897	906%
Hydrogen permeability (mol/cm-s-bar) × 10^11^	-	0.9	28%
Electro-osmotic drag (molecule H_2_O/charge carrier)	T = 30 °C	0.6	28%
	T = 80 °C	0.4	30%
